# The effects of Tai Chi on standing balance control in older adults may be attributed to the improvement of sensory reweighting and complexity rather than reduced sway velocity or amplitude

**DOI:** 10.3389/fnagi.2024.1330063

**Published:** 2024-04-08

**Authors:** Jianhua Cui, Zengming Hao, Haibo Tian, Yi Yang, Jian Wang, Xiaomei Lin

**Affiliations:** ^1^Department of Sports Science, College of Education, Zhejiang University, Hangzhou, China; ^2^Department of Rehabilitation Medicine, The First Affiliated Hospital, Sun Yat-sen University, Guangzhou, China; ^3^School of Teacher Education, Shaoxing University, Shaoxing, China; ^4^Center for Psychological Sciences, Zhejiang University, Hangzhou, China

**Keywords:** Tai Chi, older adults, standing balance, wavelet analysis, multiscale entropy, recurrence quantification analysis, sensory reweighting, complexity

## Abstract

**Introduction:**

Tai Chi has proved to be an effective therapy for balance performance and cognition. However, non-consistency exists in the results of the effect of Tai Chi training on standing balance control in older adults. This study aimed to use traditional and non-traditional methods to investigate the effect of Tai Chi on standing balance in older adults.

**Methods:**

Thirty-six Tai Chi practitioners (TC group) and thirty-six older adults with no Tai Chi practice (control group) were recruited in this study. A Nintendo Wii Balance Board was used to record the center of pressure (COP) during standing balance over 20 s in the condition of eyes closed with three repetitions. The wavelet analysis, multiscale entropy, recurrence quantification analysis, and traditional methods were used to evaluate the standing balance control in the anterior-posterior (AP) and mediolateral (ML) directions.

**Results:**

(1) Greater sway mean velocity in the AP direction and sway Path length were found in the TC group compared with the control group; (2) lower Very-low frequency band (0.10–0.39 Hz) and higher Moderate frequency band (1.56–6.25 Hz) in the AP and ML directions were found in the TC group compared with the control group; (3) greater complexity index (CI) and lower determinism (DET) in the AP and ML directions were observed in the TC group compared with control group; (4) greater path length linked with smaller Very-low frequency band in the AP and ML directions and higher Moderate frequency band in the AP direction in both groups; (5) greater path length linked with lower DET and higher CI in the AP direction only in the TC group.

**Conclusion:**

Long-term Tai Chi practice improved sensory reweighting (more reliance on the proprioception system and less reliance on the vestibular system) and complexity of standing balance control in older adults. In addition, greater sway velocity may be as an exploratory role in standing balance control of TC older adults, which correlated with greater complexity, but no such significant relationship in the control group. Therefore, the effects of Tai Chi practice on standing balance control in older adults may be attributed to the improvement of sensory reweighting and complexity rather than reduced sway velocity or amplitude.

## 1 Introduction

The world's population is aging, and one of the main problems associated with aging is the increase of fall risk. Falls and fall-related injuries pose a serious threat to the functional independence and quality of life of older adults, which are closely related to poorer balance function in older adults (Hu and Woollacott, 1994; Salzman, 2010; Montero-Odasso et al., 2022). Hence, prevention of falls and management for older adults is a major global challenge. Older adults with different levels of risk for falls should be offered education about the prevention of falls and targeted exercise (Izquierdo et al., 2021; Montero-Odasso et al., 2022). Among the targeted exercises, Tai Chi can significantly improve balance and cognitive function in older adults (Chang et al., 2014; Hackney and Wolf, 2014; Pan et al., 2018; Wang et al., 2022). However, the effects of resistance exercise, dance, or walking on the rate of falls remain uncertain (Sherrington et al., 2019). It has been found that the combination of higher visual dependence and slower down-weighting contributes to postural instability in older adults (Jeka et al., 2010), and impairment of multisensory integration could predispose older adults to falls (Zhang et al., 2020). Remarkably, long-term Tai Chi practice increased the complexity of standing balance (Wayne et al., 2014a) and improved the multisensory integration in older adults (Wang et al., 2023). Therefore, it is crucial and necessary to investigate the effects of Tai Chi on sensory reweighting and the complexity of standing balance control in older adults.

Tai Chi is a Chinese conditioning exercise and is a strong recommendation for the prevention of falls in older adults (Lan et al., 2002), which integrates balance, flexibility, and neuromuscular control along with a complex attentional process (Wayne et al., 2014a; Rath and Wade, 2017). Although different forms of exercise have different effects on preventing falls (Sherrington et al., 2019), Tai Chi is a relatively effective exercise in improving balance control and preventing falls in older adults compared with other types of exercise (Low and Walsh, 2017). Tai Chi practitioners commonly exhibited better balance and cognitive function compared to non-intervention controls (Lan et al., 2002; Wayne et al., 2014a). Moreover, although motor control deficits exist in patients with Parkinson's disease (Li et al., 2022; Smart et al., 2023) and stroke survivors (Ma et al., 2017; Chen et al., 2019), Tai Chi practice has been used successfully for improving balance function in older patients with stroke (Taylor-Piliae et al., 2014), Parkinson's disease (Li et al., 2022), and mild cognitive impairment (Sungkarat et al., 2017), which may be attributed to the improvement in neuromuscular function in older adults practicing Tai Chi (Hu et al., 2021). Nevertheless, several studies have found that non-consistency exists in the results of the effect of Tai Chi training on standing balance in older adults, demonstrating that the standing balance of older adults is affected by many factors, such as sensory functions, cognition, and fear of falling. Tai Chi practice has been shown to improve proprioception (Xu et al., 2004; Zhang et al., 2021) and cognitive function (Chang et al., 2014; Wayne et al., 2014b) and reduce the fear of falling in older adults (Wolf et al., 1997; Sattin et al., 2005). In addition, the testing conditions of standing balance and the selection of evaluating metrics are also possible reasons for inconsistency in the results of Tai Chi training on the standing balance in older adults. No differences in simple static postural control between older adults practicing Tai Chi and the control group were observed, while the older adults with regular Tai Chi practice showed better postural stability in more challenging conditions (Wong et al., 2001). Thus, the challenging testing conditions seem more likely to have significant effects of Tai Chi training on standing balance in older adults. However, challenging testing conditions were commonly associated with reduced safety in balance and fear of falling (Young and Mark Williams, 2015), which in turn influenced the control of standing balance. On the other hand, traditional measures showed small sensitivities for assessing the standing balance between older adults with and without Tai Chi practice compared to non-traditional measures (Kang et al., 2009; Wayne et al., 2014a). Therefore, it is substantial to evaluate the effect of Tai Chi training on standing balance control in older adults under moderately difficult testing conditions using non-traditional methods.

The assessment of the center of pressure (COP) data obtained via the use of a force plate was commonly used to assess the standing balance in older adults. Many traditional measures, such as sway velocity, sway area, and sway path length, were often used to evaluate standing balance (postural sway). However, these traditional measures had some unignorable limitations (Hao et al., 2021). For example, in a randomized controlled trial investigating different interventions for preventing falls in community-dwelling older people (Sturnieks et al., 2024), the rate of falls was significantly reduced in the exergame training group compared with the control group, but the path length was not statistically different between the two groups; these results demonstrated that path length cannot well-reflect the balance control and the risk of falling. In contrast, some non-traditional methods, such as multiscale entropy (Busa and van Emmerik, 2016; Zhou et al., 2017), recurrence quantification analysis (Ramdani et al., 2013; Hao et al., 2021), and other non-linear measures (Kedziorek and Błażkiewicz, 2020), have been proven to provide crucial information about COP data, thereby contributing to more comprehensive insights into the control of standing balance in older adults. In addition, it is commonly believed that overreliance on vision may be disruptive to balance control in older adults (Jeka et al., 2010). Long-term Tai Chi practitioners exhibited better-standing balance control after vestibular stimulation than older control adults (Tsang and Hui-Chan, 2006), demonstrating that long-term Tai Chi practice would significantly change the vestibular contribution of standing balance in older adults. In addition, long-term Tai Chi practice also improved the standing balance in older adults with increased reliance on the visual and vestibular systems (Tsang et al., 2004). Thus, it is very important to assess the influence of visual, vestibular, and somatosensory inputs on balance control in older adults. Notably, frequency analysis based on discrete wavelet transform can reflect different sensory contributions in standing balance control in older adults (Quek et al., 2014). Hence, these non-traditional measures mentioned may be helpful in further understanding the effects of Tai Chi on the sensory reweighting and complexity of standing balance control in older adults.

Thus, this study aimed to investigate the effect of long-term Tai Chi practice on standing balance in older adults by using traditional and non-traditional methods (frequency analysis, multiscale entropy, and recurrence quantification analysis). The hypotheses are as follows: (1) the postural stability in standing balance differs between older adults with and without long-term Tai Chi practice; (2) long-term Tai Chi practice would change the sensory reweighting and complexity in standing balance of older adults; and (3) postural sway correlated with sensory reweighting and complexity in older adults with long-term Tai Chi practice.

## 2 Materials and methods

### 2.1 Participants

The sample size was calculated using PASS 15.0.5 software based on the mean and standard deviation of the center of pressure complexity in the pilot stage of this study and in a previous study (Wayne et al., 2014a). To produce the power of 80% at an alpha level of 0.05, the sample size of 25 was required in each group. Seventy-two community-dwelling older subjects, aged 60 to 89 years, participated in this study. Thirty-six Tai Chi practitioners (TC group) were recruited from a local Tai Chi club. All of them had practiced Tai Chi for at least 5 years. Thirty-six older adults from local community elderly centers with no previous experience in Tai Chi practice (control group) were recruited to match the TC group for gender, age, weight, and height (Table 1). All the subjects were able to communicate and follow the test and provided written consent before any measurements. The exclusion criteria included taking help of walking aids, any cognitive impairments, the diagnosis of any neurological disorder, peripheral neuropathy of the lower limbs, and fall experiences in the past 12 months. This study was approved by the Research Ethics Board of the Center for Psychological Sciences at Zhejiang University (issued no.2020-003).

**Table 1:** Descriptive characteristics of the participants.

	**TC group**	**Control group**	***t***	***p***
Age (years)	74.81 (7.45)	72.06 (5.70)	1.759	0.083
Gender (male/female)	10/26	10/26		
Body weight (kg)	58.47 (10.88)	59.67 (9.46)	−0.497	0.621
Body height (cm)	158.50 (7.24)	159.58 (8.18)	−0.595	0.554
Body mass index (kg/m^2^)	23.15 (2.93)	23.35 (2.65)	−0.303	0.763
Exercise experience (years)	13.75 (4.34)	0		
Frequency (times a week)	5.68 (0.93)	0		
Duration (min per session)	74.33 (16.37)	0		

Values are expressed as mean (standard deviations) or number. TC (Tai Chi).

### 2.2 Data collection

To evaluate static standing balance control in older adults, a Nintendo Wii Balance Board (WBB; Nintendo, Kyoto, Japan) connected by bluetooth to a laptop computer was used to record COP displacements. Many research studies have demonstrated that the WBB, which is portable, low-cost, and suitable for clinical settings, can be used as a reliable and valid tool for assessing standing balance (Clark et al., 2018), especially with the condition of quiet standing (Clark et al., 2010; Bartlett et al., 2014; Leach et al., 2014). The COP signals were obtained using the software BrainBLoX with a sampling rate of 100 Hz (Cooper et al., 2014). In an upright bipedal stance, all older subjects were asked to stand quietly on the WBB with their eyes closed, keep their arms beside their bodies, and maintain standing balance by being as still as possible and wearing noise-canceling headphones designed to reduce external noise. Standing balance was measured for three trials, and the test duration was 20 s. All participants familiarized themselves with the test before formal testing and had 30 s of rest between different trials. Throughout the test, one investigator stayed close to the participants to prevent them from falling. Once the participants moved their feet, the trial was stopped, and they were excluded from further analysis. All the standing balance tests were completed in the community centers.

### 2.3 Data analysis

**Figure 1:**
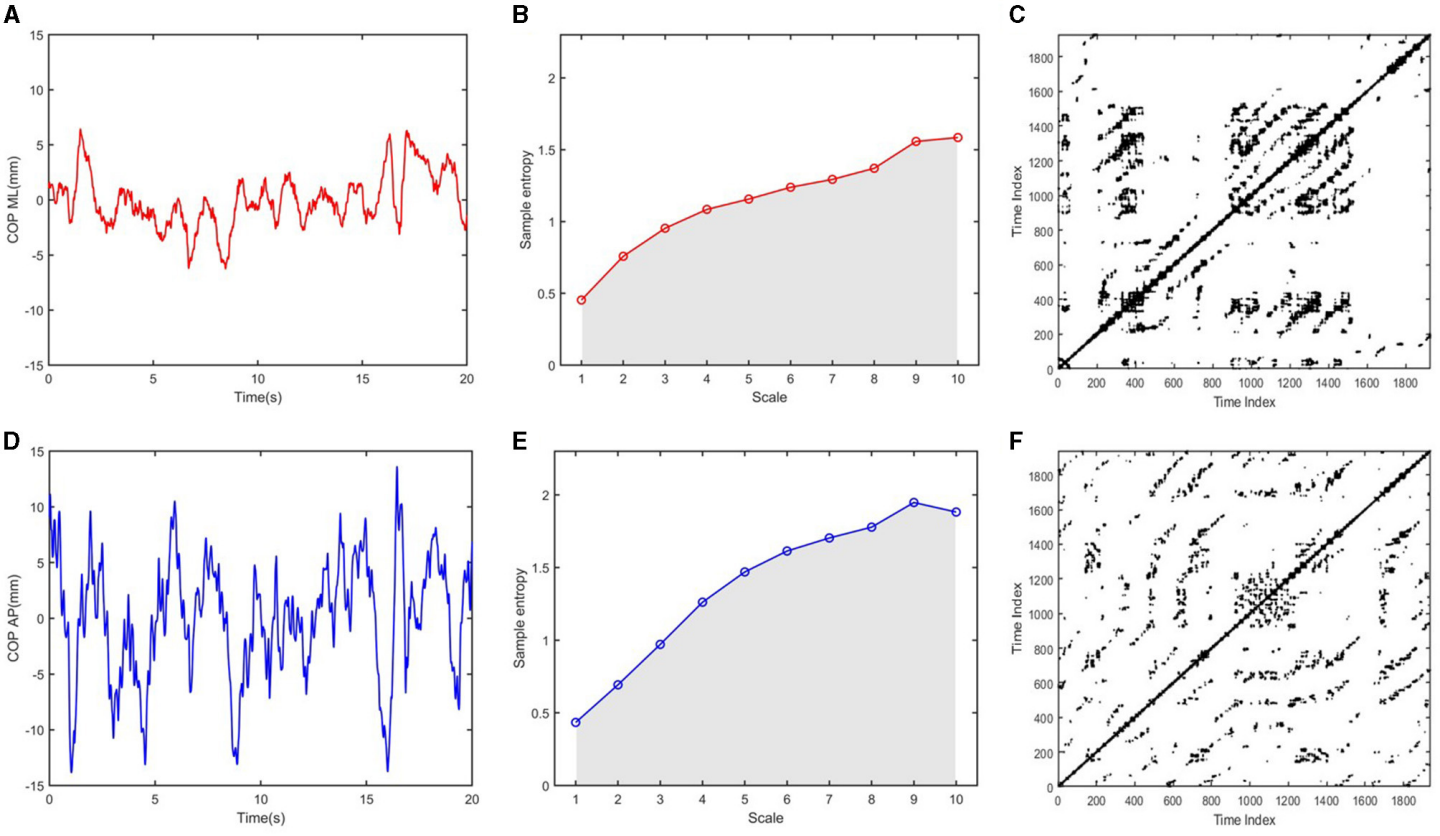
Multiscale entropy and recurrence plots generated from COP signals: **(A, D)** COP time series in the ML and AP directions; **(B, E)** Multiscale entropy for 10 time scales were calculated from COP ML and COP AP time series, respectively; The complexity index (CI) was obtained by calculating the area under the sample entropy vs. the time scale; **(C, F)** Recurrence plots were generated from COP ML and COP AP time-series, respectively.

The COP positions obtained by the WBB were filtered using a 20-Hz low-pass, second-order, zero-lag Butterworth filter. Then, the data's linear trend was removed by subtracting them from their mean (Figures 1A, D). Furthermore, the COP displacements in the anterior–posterior (AP) and medial–lateral (ML) directions were analyzed using both traditional and non-traditional methods. Traditional methods included range, standard deviation, sway mean velocity, sway path length, and sway area, which were used in our previous study (Hao et al., 2021). The range is the distance between the maximum and minimum COP displacement in the AP and ML directions. SD is defined as the square root of the mean of the squares of COP displacement in the AP and ML directions. Sway mean velocity (MV) is calculated by dividing the COP excursion in the AP and ML directions by the duration time. Path length quantifies the magnitude of the two-dimensional displacement based on the total distance traveled. Sway area (area) quantifies 85% of the total area covered in the ML and AP directions by using an ellipse to fit the COP data. It is generally believed that the smaller the value of these traditional measures of COP signals, the better the balance performance. In addition, the non-traditional methods in our study included frequency analysis, multiscale entropy (MSE), and recurrence quantification analysis (RQA).

Frequency analysis of COP displacements was performed using discrete wavelet transform (Quek et al., 2014). The COP signal was split into four frequency bands using a 12-level Symlet-8 wavelet (St-Amant et al., 2020; Lafleur and Lajoie, 2023): (1) ultralow frequency band (< 0.10 Hz), (2) very-low frequency band (0.10–0.39 Hz), (3) low-frequency band (0.39–1.56 Hz), and (4) moderate-frequency band (1.56–6.25 Hz). These frequency bands were calculated and represented in terms of the percentage of total energy (Lin et al., 2019), which were believed to gain postural movements associated with the visual system (ultralow), the vestibular system (very-low), the cerebellar system (low), and muscular proprioception (moderate), respectively.

Multiscale entropy (MSE) was used to assess the degree of irregularity in the fluctuation of the COP signal at multiple time scales (Busa and van Emmerik, 2016). First, empirical mode decomposition was used to remove relatively low-frequency components (< 0.2 Hz) from the COP signal. Second, filtered time series were then “coarse-grained” to capture system dynamics on a given time scale. As 2, 000 data points were available for the MSE analysis and 200 data points per window are required to elicit consistent sample entropy values, we calculated the sample entropy values for 10 time scales to determine the degree of irregularity using an m of 2 and an r of 15% of the standard deviation of the time series. Finally, the complexity index (C_I_) was obtained by calculating the area under the sample entropy vs. time-scale curve (Figures 1B, E). Consequently, a higher complexity index indicated greater irregular and unpredictable sway (Manor et al., 2010).

Recurrence quantification analysis (RQA) was used to assess the dynamics of the COP signal based on the construction of a recurrence plot (van den Hoorn et al., 2018; Hao et al., 2021). The time delay and the embedded dimension were calculated using the mutual information method and false nearest neighbor analysis, respectively (Marwan et al., 2007). Recurrence plots were generated according to a preset threshold (Figures 1C, F). Determinism (DET) refers to the percentage of all recurrences in the phase space that forms diagonal lines with lengths longer than a pre-set threshold distance. Higher DET values indicate a more predictable, regular COP signal. The recurrence threshold was chosen as 5% of the recurrence rate, and the minimal length of the diagonal line was set as 100 ms.

### 2.4 Statistics

All statistical testing was conducted using SPSS (IBM SPSS Statistics, Version 25, SPSS Inc., Chicago, IL, USA). Each measure's distribution of normality was tested (Shapiro–Wilk test, *p* > 0.05). Independent *t*-tests (normally) or Mann–Whitney U tests (non-normally) were used to compare all COP measures between the TC group and the control group. Because the global path length is commonly used to represent the balance control (postural sway), and the abnormality in postural sway has been associated with various clinical outcomes including fall risk, Spearman's correlations were computed to determine the relationship between path length and non-traditional measures (frequency and complexity measures) of COP data in the TC and control groups, respectively. The significance level was set as a *p* < 0.05 with a two-tailed test. Effect size values (Cohen's d for independent *t*-tests and rank biserial correlation coefficient for Mann–Whitney U tests) were reported. Thresholds of 0.20, 0.50, and 0.80 for Cohen's d and thresholds of 0.10, 0.30, and 0.50 for the correlation coefficient signify small, moderate, and large effect sizes, respectively (Cohen, 1988).

## 3 Results

### 3.1 Traditional measures

Table 2 provides the result of traditional measures between the TC group and the control group. The TC group exhibited significantly higher MV_ap and path length than the control group. Small-to-moderate effect sizes were found for these traditional measures (Table 2).

**Table 2:** Comparison of traditional measures of standing balance between the TC group and control group.

	**TC group**	**Control group**	***p***	**ES**
Range_ml (mm)	10.34 (4.73)	14.00 (11.63)	0.139	−0.204
Range_ap (mm)	28.49 (10.27)	31.10 (13.86)	0.634	−0.066
SD_ml (mm)	1.97 (0.88)	2.70 (2.06)	0.215	−0.171
SD_ap (mm)	5.32 (1.46)	6.31 (2.72)	0.155	−0.196
MV_ml (mm/s)	12.31 (3.49)	12.81 (6.78)	0.520	0.090
MV_ap (mm/s)	23.07 (12.50)	18.91 (13.89)	**0.024**	0.309^#^
Path length (mm)	571.76 (256.10)	509.60 (308.97)	**0.045**	0.275
Area (mm^2^)	206.32 (141.98)	347.36 (483.09)	0.250	−0.159

Values are expressed as mean (standard deviations); TC (Tai Chi); ES (effect size). Bold p-values represent p < 0.05. # denotes an effect size greater than the moderate threshold (0.3) for Mann–Whitney U tests.

### 3.2 Frequency and complexity measures

Table 3 provides the result of frequency and complexity measures between the TC group and the control group. Statistically significant differences were observed for the very-low-frequency band, the moderate-frequency band, CI, and DET in the both AP and ML directions. The TC group showed significantly lower very-low_ml frequency band, very-low_ap frequency band, DET_ml, and DET_ap than the control group, whereas higher moderate_ml frequency band, moderate_ap frequency band, CI_ml, and CI_ap were observed compared with the control group. Medium-to-large effect sizes were found for these measures (Table 3).

**Table 3:** Comparison of non-traditional measures of standing balance between the TC group and control group.

	**TC group**	**Control group**	***p***	**ES**
Ultralow_ml (%)	11.02 (5.26)	11.99 (6.08)	0.534	−0.086
Ultralow_ap (%)	9.73 (4.85)	10.73 (5.23)	0.626	−0.068
Very-low_ml (%)	17.98 (5.21)	24.06 (9.76)	**0.006**	−0.372^#^
Very-low_ap (%)	24.48 (8.70)	29.41 (9.21)	**0.022**	−0.551^†^
Low_ml (%)	43.96 (6.93)	41.69 (10.04)	0.268	0.263
Low_ap (%)	39.56 (6.64)	38.81 (7.86)	0.663	0.103
Moderate_ml (%)	27.04 (6.28)	22.27 (5.98)	**< 0.001**	0.454^#^
Moderate_ap (%)	26.23 (8.03)	21.05 (7.57)	**0.004**	0.387^#^
C_I__ml	12.26 (2.32)	10.47 (3.56)	**0.014**	0.593^†^
C_I__ap	10.60 (2.47)	8.29 (2.29)	**< 0.001**	0.971^†^
DET_ml	72.87 (10.35)	77.85 (13.69)	**0.017**	−0.326^#^
DET_ap	79.55 (11.27)	88.05 (8.05)	**< 0.001**	−0.497^#^

Values are expressed as mean (standard deviations); TC (Tai Chi); ES (effect size). Bold p-values represent p < 0.05. ^†^ denotes an effect size greater than the medium threshold (0.5) for independent t-tests. ^#^ denotes an effect size greater than the moderate threshold (0.3) for Mann–Whitney U tests.

### 3.3 Relationships between path length and non-traditional measures in the TC group and control group

Statistically significant relationships were observed between path length and non-traditional measures in the TC group and control group (Table 4). For frequency measures, path length was negatively correlated with very-low_ml and very-low_ap and positively correlated with moderate_ap in both groups. For complexity measures, path length was negatively correlated with DET_ap and positively correlated with CI_ap within the TC group, while no significant correlation was found in the control group.

**Table 4:** Correlations of path length and non-traditional measures in the TC group and control group.

	**TC group**		**Control group**	
	**Path length**		**Path length**	
**Variables**	***r***	***p***	***R***	***P***
Ultralow_ml	−0.251	0.140	−0.366	0.029
Ultralow_ap	−0.447	0.007	−0.200	0.242
Very-low_ml	−0.524	**0.001**	−0.516	**0.001**
Very-low_ap	−0.759	**< 0.001**	−0.460	**0.005**
Low_ml	0.239	0.160	0.392	0.019
Low_ap	0.395	0.018	0.104	0.545
Moderate_ml	0.278	0.100	0.403	0.015
Moderate_ap	0.784	**< 0.001**	0.505	**0.002**
CI_ml	0.042	0.808	0.124	0.469
CI_ap	0.683	**< 0.001**	0.240	0.157
DET_ml	−0.059	0.731	−0.137	0.423
DET_ap	−0.784	**< 0.001**	−0.485	0.003

r, correlation coefficient. Bold p values represent p < adjusted significance level (0.05/12). Shaded cells denote significant Spearman's correlation coefficient.

## 4 Discussion

The main findings are as follows: (1) greater sway mean velocity in the AP direction and sway path length were found in the TC group than in the control group; (2) lower very-low frequency band (0.10–0.39 Hz) and higher moderate-frequency band (1.56–6.25 Hz) in the AP and ML directions were found in the TC group when compared with the control group; (3) greater complexity index (CI) and lower determinism (DET) in the AP and ML directions were observed in the TC group when compared with the control group; (4) greater path length linked with smaller very-low frequency band (very-low_ml and very-low_ap) and higher moderate-frequency band (Moderate_ap) in both groups; (5) greater path length linked with lower DET_ap and higher CI_ap only in the TC group. These results are discussed below in detail.

### 4.1 Increased sway velocity in the TC group

Many previous studies suggest that long-term Tai Chi practice promotes better control of standing balance through the reduction of sway velocity or sway amplitude. In contrast, the present results indicated that long-term Tai Chi caused increased sway speed (MV_ap and path length), which was not consistent with the observations of some previous studies. However, the non-significant relative higher MV_ap and path length were found for the Tai Chi group in a previous study (Wayne et al., 2014a). No significant effects of long-term Tai Chi on traditional measures of COP data were also found in other studies. For example, Tai Chi intervention for 15 weeks did not improve the traditional measures of postural stability in older adults, but less fear of falls after training was found in older subjects in the intervention group compared with older subjects in the control group (Wolf et al., 1997). In fact, no lower fall risk in older adults after Tai Chi training was observed (Logghe et al., 2009), which demonstrated that the association between standing balance and fall risk in older adults is complicated. In addition, no significant difference was also found in the anteroposterior and mediolateral body sway during static standing balance in older adults with (mean experience = 10.1 years) and without Tai Chi practice experience, whereas older adults with Tai Chi practice showed better knee joint proprioception acuity and better limits of stability than the control group (Tsang and Hui-Chan, 2003). Another study found no difference in sway velocity and sway area during a static double-leg stance between the Tai Chi group and non-Tai Chi group, while lower sway velocity and sway area during a single-leg stance were observed in the Tai Chi group (Mak and Ng, 2003). It is particularly noteworthy that the balance performance of older adults who had practiced Tai Chi for a long term (an average of 24.5 years) was not significantly different from the control group without Tai Chi practice during the static stance. These studies suggest that traditional measures have limitations in reflecting the effect of Tai Chi practice on balance control in older adults. In addition, more experienced Tai Chi practitioners were found to correlate with less sway in the ML direction in the single-leg stance but greater sway velocity in the double-leg stance (Mak and Ng, 2003). Nevertheless, it has also been found that the improved balance performance from 4-week intensive Tai Chi training was comparable to that of experienced Tai Chi practitioners (Tsang and Hui-Chan, 2004). It can be observed that the relationship between the experience duration of Tai Chi practice in older adults and standing balance is also complex. Therefore, increased sway velocity in the TC group in our study may be the consequence of different control strategies during standing balance in older adults with and without long-term Tai Chi practice, and it may be difficult to discriminate these differences with the traditional measures only.

### 4.2 Reweighting of the sensory inputs for postural control in the TC group

In this study, we decomposed the energy content of COP signals into four distinct frequency bands, which represent the visual, vestibular, cerebellar, and proprioceptive systems. According to the result of frequency analysis, the higher moderate-frequency band and lower very-low-frequency band were observed in the TC group, which demonstrated that the TC group relies on more proprioception and less on the vestibular system in standing balance control than the control group. It has been found that older adults utilize vestibular regulation as the primary postural strategy in dual-task standing compared with young adults (Lee et al., 2020), which demonstrated that older adults adopted the control strategy with larger vestibular contribution in standing balance compared with young adults. In addition, older patients with neck pain relied more on the vestibular system and less on proprioception in standing balance control with and without vision than older adults with no neck pain (Quek et al., 2014), patients with cervical spondylotic myelopathy exhibited lower proprioceptive contribution and higher vestibular contribution under the eyes-open stance (Lin et al., 2019), and older adults with long-term Tai Chi practice showed better balance control after vestibular stimulation than the older control group (Tsang and Hui-Chan, 2006). These results demonstrated that neck pain or disease significantly enhanced the vestibular contribution in standing balance control, while long-term Tai Chi practice significantly changed the control strategy with improved proprioception in standing balance control (Xu et al., 2004; Shao et al., 2022). However, no differences in the ultralow frequency band and low-frequency band were found between the two groups, which showed that long-term Tai Chi practice did not change the visual weight significantly in standing balance control. It has been found that older women with and without neck pain showed no difference in visual contribution in standing balance control (Quek et al., 2014). In fact, Tai Chi practitioners had better balance control after vestibular stimulation (Tsang and Hui-Chan, 2006). Superior visuospatial ability and postural stability were also found in older adults with long-term Tai Chi practice, which was associated with sensitive proprioception and tactile sensation (Shao et al., 2022). Therefore, long-term Tai Chi practice significantly improved sensory reweighting in standing balance control possibly through enhanced proprioceptive contribution and less vestibular contribution.

### 4.3 Greater complexity of postural control in the TC group

Increased irregularity is commonly a sign of automaticity and better control of standing balance (Donker et al., 2007), which was consistent with present findings. Higher C_I_ and lower DET in the Tai Chi group in this study demonstrated increased irregularity and decreased regularity, respectively (Busa and van Emmerik, 2016). The index of C_I_ has been proven to be a better predictor of future falls in older adults (Zhou et al., 2017), while traditional measures did not predict future falls. However, it has been found that C_I_ did not distinguish older adults with risk of falling from those not at risk offalling, but higher DET was significantly observed in fallers (Ramdani et al., 2013), which was consistent with the result in our study that observed higher DET in the control group compared with the Tai Chi group. Based on the results of complexity analysis in this study, multiscale entropy and recurrence quantification analysis are promising approaches for the evaluation of COP fluctuations in older adults. In addition, it has been found that both visual impairment and somatosensory impairment decreased the complexity index of standing balance in older adults and impaired feedback control related to low physiological complexity (Manor et al., 2013). Therefore, it is substantial and necessary to evaluate postural control using complexity measures, especially for understanding the dynamics of standing balance in older adults. It is worth noting that older adults with long-term Tai Chi training showed greater complexity of standing balance than older adults without Tai Chi practice, and increased complexity among those older adults randomized to the Tai Chi training group was positively correlated with practice hours (Wayne et al., 2014a). Tai Chi training increased the complexity index of standing balance in older adults with peripheral neuropathy, while traditional measures of COP signals did not change significantly from the baseline (Manor et al., 2013). In fact, both MSE and RQA can reflect the complexity, and RQA is more sensitive in distinguishing the standing balance control of non-fallers and fallers compared with MSE (Ramdani et al., 2013). Thus, different complexity measures of COP signals may be more suitable to reflect on the effect of short-term and long-term Tai Chi practice on standing balance control in older adults.

### 4.4 Greater path length linked with lower vestibular and higher proprioception contribution in both groups

According to the results of relationships between path and frequency measures in both groups, path length was negatively correlated with very-low-frequency band (Very-low_ml and Very-low_ap) and positively correlated with moderate-frequency band (Moderate_ap) in both groups, which demonstrated that greater sway velocity was linked with lower vestibular and higher proprioception contribution in both groups. Combining these relationships above and significant differences between the very-low-frequency band and moderate-frequency band between the *two* groups partially explained the trends toward smaller postural sway velocity in the control group and greater postural sway velocity in the Tai Chi group, which was mentioned before in a previous study (Wayne et al., 2014a). Therefore, postural sway in both groups was linked with the vestibular and proprioceptive contribution of standing balance, which suggested that older adults with long-term Tai Chi practice may use greater postural sway in standing balance as an exploratory strategy to ensure continuous dynamic inputs from multiple sensory systems (Carpenter et al., 2010; Murnaghan et al., 2011). In fact, it has been proven that long-term Tai Chi practice can significantly change the control strategy compared to the control group (Huang et al., 2022; Shao et al., 2022). On the contrary, older adults with Parkinson's disease (PD) exhibited shorter path length compared to older adults without PD during the unipedal stance, which was explained as the patients with PD are unable to use the exploratory strategy (Smart et al., 2023). In addition, it can be observed that traditional measures can provide limited information for assessing standing balance in older adults.

### 4.5 Greater path length linked with higher complexity of postural control in the TC group

According to the results of relationships between path length and complexity measures in both groups, it was found that path length was not significantly correlated with any complexity measures in the AP and ML directions in the control group, which was consistent with the findings in the previous studies (Kang et al., 2009; Manor et al., 2010). However, greater path length was associated with a higher complexity index and lower determinism in the AP direction for the Tai Chi group. Different correlations between path length and complexity measures in the *two* groups may account for the difference in the control strategy between the Tai Chi group and the control group. In fact, complexity measures have been proven to be a better predictor of future falls in older adults compared with the traditional measures (Zhou et al., 2017), and different complexity measures have distinct sensitivities for distinguishing the standing balance between non-fallers and fallers (Ramdani et al., 2013). Therefore, postural sway in the Tai Chi group correlated with the complexity measures in the AP direction, which suggested that older adults with long-term Tai Chi practice may use greater postural sway in standing balance as an exploratory strategy (Carpenter et al., 2010; Murnaghan et al., 2011).

### 4.6 Limitations

This study only used the center of pressure obtained from the Nintendo Wii Balance Board to assess sensory reweighting and the complexity of standing balance in the Tai Chi group and control group, and the effects of long-term Tai Chi practice on muscular and cerebral activity in older adults need to be investigated in future. In addition, the condition with eyes closed under a firm surface was only used in this study, without considering the effect of more challenging testing conditions or cognition tasks on the balance control in older adults.

### 4.7 Future recommendations

Based on the existing significant differences in standing balance control reflected by multiple measures of COP data between the TC group and control group in this study, assessing the sensory reweighting and complexity rather than just sway velocity or amplitude in the standing balance is recommended, and the central mechanism and neuromuscular control mechanism of Tai Chi practice in improving balance function in older adults are also needed to be explored in future work. In addition, Tai Chi practice has been proven to effectively improve balance function for older patients with stroke, Parkinson's disease, and mild cognitive impairment. However, path length or other traditional measures only cannot well evaluate the balance control and the risk of falling in older adults. Thus, for investigating the effect of short-term and long-term Tai Chi practice on balance control in older patients with Parkinson's disease, stroke, and mild cognitive impairment, the evaluation of sensory reweighting and complexity may provide significant insights into the balance function assessments and effective treatments for these older patients.

## 5 Conclusion

Long-term Tai Chi practice improved sensory reweighting (more reliance on the proprioception system and less reliance on the vestibular system) and complexity of standing balance control in older adults. In addition, greater sway velocity may have an exploratory role in standing balance control of TC older adults, which correlated with greater complexity, but no such significant relationship was observed in the control group. Therefore, the effects of Tai Chi on standing balance control in older adults may be attributed to the improvement of sensory reweighting and complexity rather than reduced sway velocity or amplitude.

## Data availability statement

The raw data supporting the conclusions of this article will be made available by the authors, without undue reservation.

## Ethics statement

The studies involving humans were approved by Research Ethics Board of Center for Psychological Sciences at Zhejiang University. The studies were conducted in accordance with the local legislation and institutional requirements. The participants provided their written informed consent to participate in this study. Written informed consent was obtained from the individual(s) for the publication of any potentially identifiable images or data included in this article.

## Author contributions

JC: Conceptualization, Investigation, Methodology, Project administration, Resources, Writing – original draft, Writing – review & editing. ZH: Conceptualization, Data curation, Formal analysis, Investigation, Methodology, Software, Visualization, Writing – original draft, Writing – review & editing. HT: Data curation, Investigation, Methodology, Writing – review & editing. YY: Data curation, Investigation, Methodology, Writing – review & editing. JW: Conceptualization, Investigation, Methodology, Supervision, Validation, Writing – review & editing. XL: Conceptualization, Funding acquisition, Investigation, Methodology, Project administration, Resources, Software, Supervision, Validation, Writing – review & editing.
